# Fetal Exposure to Perfluorinated Compounds and Attention Deficit Hyperactivity Disorder in Childhood

**DOI:** 10.1371/journal.pone.0095891

**Published:** 2014-04-23

**Authors:** Amanda Ode, Karin Källén, Peik Gustafsson, Lars Rylander, Bo A. G. Jönsson, Per Olofsson, Sten A. Ivarsson, Christian H. Lindh, Anna Rignell-Hydbom

**Affiliations:** 1 Division of Occupational and Environmental Medicine, Lund University, Lund, Sweden; 2 Child and Adolescent Psychiatry Unit, Department of Clinical Sciences, Lund University, Lund, Sweden; 3 Obstetrics and Gynecology Unit, Department of Clinical Sciences, Skåne University Hospital, Lund University, Malmö, Sweden; 4 Department of Clinical Sciences, Unit of Pediatric Endocrinology, Lund University/Clinical Research Centre (CRC), Malmö, Sweden; University of Cincinnati, United States of America

## Abstract

**Background:**

The association between exposure to perfluorinated compounds (PFCs) and attention deficit hyperactivity disorder (ADHD) diagnosis has been sparsely investigated in humans and the findings are inconsistent.

**Objectives:**

A matched case-control study was conducted to investigate the association between fetal exposure to PFCs and ADHD diagnosis in childhood.

**Methods:**

The study base comprised children born in Malmö, Sweden, between 1978 and 2000 that were followed up until 2005. Children with ADHD (n = 206) were identified at the Department of Child and Adolescent Psychiatry. Controls (n = 206) were selected from the study base and were matched for year of birth and maternal country of birth. PFC concentrations were measured in umbilical cord serum samples. The differences of the PFC concentrations between cases and controls were investigated using Wilcoxon's paired test. Possible threshold effects (above the upper quartile for perfluorooctane sulfonate (PFOS) and perfluorooctanoic acid (PFOA) and above limit of detection [LOD] for perfluorononanoic acid (PFNA)) were evaluated by conditional logistic regression.

**Results:**

The median umbilical cord serum concentrations of PFOS were 6.92 ng/ml in the cases and 6.77 ng/ml in the controls. The corresponding concentrations of PFOA were 1.80 and 1.83 ng/ml. No associations between PFCs and ADHD were observed. Odds ratios adjusted for smoking status, parity, and gestational age were 0.81 (95% confidence interval [CI] 0.50 to 1.32) for PFOS, 1.07 (95% CI 0.67 to 1.7) for PFOA, and 1.1 (95% CI 0.75 to 1.7) for PFNA.

**Conclusions:**

The current study revealed no support for an association between fetal exposure to PFOS, PFOA, or PFNA and ADHD.

## Introduction

Emission of pollutants from densely populated areas and industries is a growing environmental problem. Contaminants present in the environment can have a negative impact on both human health and environment. Perfluorinated compounds (PFCs) are extremely stable and persistent man-made organic chemicals that have been identified as environmental pollutants. The unique properties of PFCs have made them highly useful in numerous industrial and consumer applications such as lubricants, firefighting foams, cleaning agents, and in surface coating for paper, food packaging, textiles, furniture, carpets and cookware [1]–[3].

PFCs, particularly perfluorooctane sulfonate (PFOS) and perfluorooctanic acid (PFOA) have been widely detected in the environment, wildlife, and humans [4]–[8]. Humans are exposed to PFCs through consumer products as well as contaminated air, water, and food [1]. In recent years, studies have revealed that PFCs cross the placenta and accumulate in the fetus [9]–[11]. The fetal brain is immature and is therefore susceptible to injury caused by toxic agents [12]. Animal data have indicated that PFCs accumulate in the brain both before and after the blood-brain barrier is formed [13]–[16].

Animal studies have shown that neonatal exposure to low doses of PFCs induced irreversible neurotoxic effects in adult mice and caused changes in behavior and habituation by altering the dopaminergic and cholinergic system [17], [18]. PFCs also alter levels of neural proteins that are important for the formation and growth of the synapses [19]. Defects in the dopamine transporters and receptors have been suggested to be the most significant neurobiological problem in attention deficit hyperactivity disorder (ADHD) [20], [21].

ADHD is a neurodevelopmental disorder defined by inattention, hyperactivity and impulsivity [22], [23]. The disorder has its onset in childhood, and persists into adolescence and into adulthood in some cases [24], [25]. The genetic factor is believed to play the major role in the development of ADHD [22], [26], [27]. In addition, exposure to environmental toxins, such as lead, mercury, and persistent chlorinated biphenyls, has also been related to ADHD [26], [28], [29].

Two cross-sectional studies based on parent-reported ADHD diagnosis have investigated the potential association between PFC levels in school-age children and ADHD [30], [31]. The study by Hoffman et al. [30] found a positive relationship between ADHD and PFC levels in the blood of children between 12 and 15 years, whereas an association with only perfluorohexane sulfonate (PFHxS) was found in the study by Stein and Savitz [31]. In another cross-sectional study, PFC exposure was associated with impulsivity in children [32]. Other studies based on questionnaires investigated whether behavioral health and motor coordination as well as motor and mental developmental milestones were associated with maternal PFCs during pregnancy and found no such associations except for PFOS which was associated with delayed motor development in the first two years of life [33], [34].

The frequency of children receiving an ADHD diagnosis has increased in recent years [35]. Improved diagnostic criteria might be responsible for the increased detection of ADHD cases. Increased exposure to environmental pollutants might also contribute to the high prevalence of ADHD. Since the human brain is susceptible to disturbance by environmental pollutants during the fetal period, it is of importance to investigate the association between exposure to these pollutants during the sensitive period of fetal development and ADHD.

The objective of this study is to investigate the association between fetal exposure to PFCs and ADHD diagnosis in childhood. Unlike previous studies, this case-control study is based on clinical ADHD diagnosis and PFCs are measured in umbilical cord serum samples which reflect the PFC concentrations in the fetus. The study is a part of the Fetal Environment and Neurodevelopment Disorders in Epidemiological Research project (the FENDER project).

## Material and Methods

### Participants

The selection procedure of the children with ADHD diagnosis has been previously described by Gustafsson and Kallen [36]. Briefly, at the Department of Child and Adolescent Psychiatry in the city of Malmö, 419 children born and living in Malmö between 1978 and 2000 with ADHD diagnosis were identified and were followed up until 2005. During the study period, the children with ADHD were diagnosed by one of ten experienced clinicians at the department using the Diagnostic and Statistical Manual of Mental Disorders (DSM). A child with suspected attention difficulties, hyperactivity and/or difficulties with impulse control is usually assessed to the child and adolescent psychiatry by a special teacher and a school psychologist or by the parents. The assessment begins with gathering information about the child's general medical health condition and the child's development from birth until the present time. The school psychologist or the psychologist at the psychiatric clinic performs a cognitive testing with the Wechsler Intelligence Scale (WISC). The parents and the teacher are asked to fill in questionnaires like SNAP-IV, Conner's questionnaire or the 5–15 questionnaire which all cover the symptoms of ADHD. Parents are usually asked to fill in the BRIEF-questionnaire concerning the child's executive functions in every-day life. Sometimes a member of the team at the clinic observes the child at school. The child's ability to concentrate is tested with TEA-Ch or with a computerized test of attention such as QB-Tech or IVA+. The child psychiatrist performs a paediatric examination with assessment of neurological soft-signs. The child's behaviour in different test situations and at the visits at the clinic is observed and registered. When all parts of the assessment have been performed, a team consisting of doctor, psychologist and sometimes a social worker meet and discuss the findings to come to a consensus decision concerning the diagnosis using DSM criteria. The DSM criteria DSM-III-R_11_ and DSM-IV_12_ were used before 1994 and from 1994 and onwards, respectively. Age at the time of diagnosis varied between 5 and 17 years, with most children being diagnosed between the ages of 8 and 12 years.

**Figure 1:**
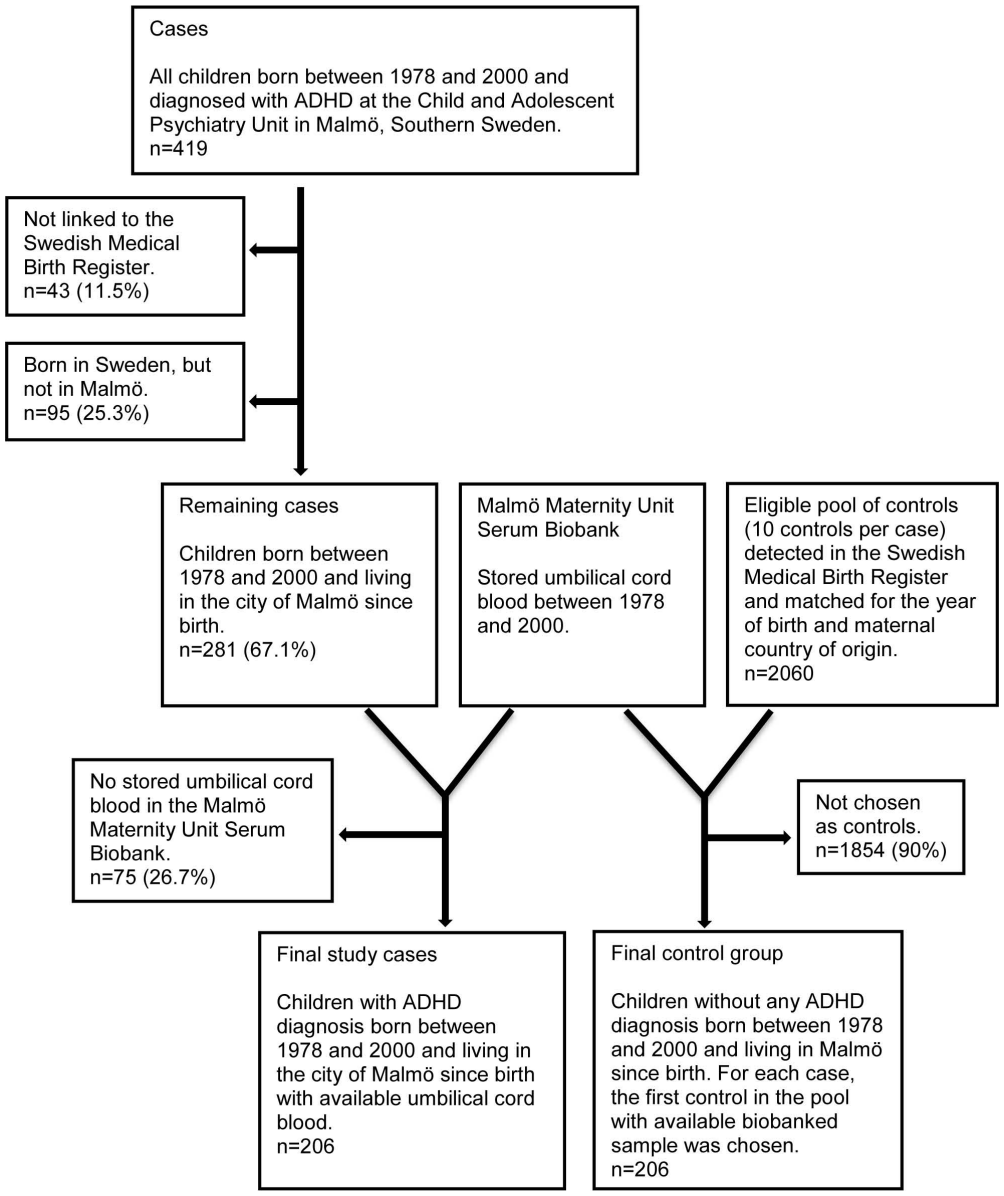
Flowchart for the selection procedure of the children with attention deficit hyperactivity disorder and controls.

Using the personal identification numbers, children with ADHD were linked to the Swedish Medical Birth Register (SMBR) which contains demographic and obstetric information on nearly all (99%) the mothers and the infants in Sweden. Umbilical cord serum samples for children with ADHD were collected from the Malmö Maternity Unit Serum Biobank (MMUSB) using the personal identification numbers. Nearly all deliveries in Malmö take place at the Malmö University Hospital Maternity Unit, where blood samples from the mother and from the umbilical cord of the newborn have been collected at the time of delivery and stored at −20°C at the MMUSB since 1969. Controls were selected into two phases. In the first phase, for each ADHD case with an available umbilical blood sample in the biobank, the next-baby-born with serum sample of the same sex was selected as a control. However, a new publication by Gustafsson and Kallen [36] revealed the impact of maternal country of birth on the diagnosis of ADHD. Thus, the benefit of matching for the maternal country of birth completely overrode that from matching for the infant's sex. Therefore, in the second phase, a pool of ten eligible controls per ADHD case were collected from the SMBR and were matched to the cases for year of birth (±12 months) and country of birth of the mother. The sample of the next-baby-born from the first phase was used if no newborn in that eligible pool of controls had an available umbilical blood sample in the biobank. The selection procedure for cases and controls is presented in Figure 1.

### Ethics statements

At the Maternity Unit, the women were informed that the umbilical cord serum sample collected could be used for research purposes in the future and those who accepted gave their verbal informed consent that was documented in the medical records. During the study period only verbal informed consent was obtained. The written informed consent has been implemented in 2005 and therefore could not be considered for the current study. The data were analysed anonymously. The study protocol followed the requirements of the Declaration of Helsinki and the study, together with the consent procedure, was approved by the Research Ethics Committee at Lund University, Sweden.

### Analysis of perfluorinated compounds and cotinine in umbilical cord serum

The analyses of PFHxS, PFOS, PFOA, PFNA, perfluorodecanoic acid (PFDA), perfluoroundecanoic acid (PFUnDA), perfluorododecanoic acid (PFDoDA), and cotinine were performed as previously described [37]. Briefly, aliquots of 100 µL sera were added with isotopically labeled internal standards, the proteins were precipitated by acetonitrile and centrifugation, and analysis was then performed using a hybrid triple quadrupole linear ion-trap mass spectrometer (LC/MS/MS; UFLCXR, Shimadzu Corporation, Kyoto, Japan; QTRAP 5500; Sciex, Framingham, MA, USA). The limits of detections for the detected PFCs and cotinine were 0.2 ng/ml. To increase the accuracy, the result reported is the average of two measurements from the same sample worked up and analyzed on different days. In all sample batches, the quality of the measurements was controlled by analyzing chemical blanks and in-house quality control (QC) samples. The reproducibility, determined as the relative standard deviation, between measured duplicate samples was 11% for PFOS, 12% for PFOA, 12% for PFNA, and 9% for cotinine. The reproducibility in QC samples was 8% for PFOS, 11% for PFOA, 8% for PFNA, and 5% for cotinine. Usually we are able to analyze several more PFCs with the method but some factor, probably during the storage of the samples, resulted in a high background noise in the chromatograms making detection impossible. Thus, PFHxS, PFDA, PFUnDA, and PFDoDA could not be detected in the samples due to this effect. On the other hand, due to the high correlation between PFOS and other PFCs often only PFOS and PFOA are reported in studies of PFCs. Although contamination of samples during collection is believed to be minimal, field blanks could not be provided to control for eventual contamination of the samples with PFCs. The analyses of PFOS and PFOA are part of the round robin intercomparison program (Professor Dr. med. Hans Drexler, Institute and Outpatient Clinic for Occupational, Social and Environmental Medicine, University of Erlangen-Nuremberg, Germany) with results within the tolerance limits.

### Statistical analyses

The Wilcoxon's paired test was used to compare the PFC concentrations between ADHD cases and controls. Conditional logistic regression analysis was used to assess the association between fetal exposure to PFCs and ADHD. The odds ratio was calculated for both 1 unit increase (nanogram per milliliter) in the concentrations of PFOS and PFOA and for comparisons between concentrations above and below the 75^th^ percentile for the control group. For PFNA the concentrations above the limit of detection (LOD) were compared to those below LOD (0.2 ng/ml).

The potential confounding variables that were considered in the present study were smoking during pregnancy, parity, and gestational age at birth, since they have been found to be associated with both PFC exposure and ADHD [11], [38]–[46].

Smoking during pregnancy was determined by cotinine levels in umbilical cord serum. Cotinine levels below the LOD (0.2 ng/ml) were related to nonsmoking pregnant women, cotinine levels higher than 15 ng/ml were related to active smokers and levels between 0.2 and 15 ng/ml were related to second-hand smokers [47]. Parity was divided into three groups according to number of previously born children (0 [i.e. nulliparous], 1, or ≥2 children). Gestational age was entered in the analyses as class variable divided into three groups; <37, 37–42, and >42 weeks of pregnancy.

The odds ratios were calculated in paired samples (n = 202) using Egret for Windows 2.0 (Cytel Software Corporation). The rest of the analyses were performed in IBM SPSS Statistics version 20 (IBM Corporation 1989, 2011).

The power calculation was based on 206 cases and matched controls. With the current setting, we had an 80% chance of detecting a difference in the levels of 0.20 standard deviations, with α value of 0.05, between cases and controls. For the analysis of the threshold effect, with α value of 0.05 and β value of 0.80, the lowest detectable odds ratio was 1.8.

## Results

PFOS and PFOA concentrations were above the LOD in 98% of the samples, whereas for PFNA about 12% were above the LOD. PFOS and PFOA concentrations below the LOD in individual samples (n = 2 for each) were replaced with 0.2 ng/ml.

The demographic characteristics and the umbilical cord PFC concentrations of the study population are presented in Table 1.

**Table 1:** Median concentration (in nanograms/milliliters) of perfluorinated compounds by the maternal and infant demographic characteristics.

Characteristics	Children with ADHD			Control group		
	n (%)	PFOS	PFOA	n (%)	PFOS	PFOA
**Group (cases/controls)**	203 (49.8%)	6.92	1.80	205 (50.2%)	6.77	1.83
**Year of delivery**						
1978–1981	2 (1.0)	2.66	0.45	2 (1.0)	8.70	0.85
1982–1985	13 (6.4)	5.69	1.50	10 (4.9)	6.49	1.71
1986–1989	63 (31.0)	6.96	2.0	66 (32.2)	6.71	1.82
1990–1993	86 (42.4)	7.08	1.78	87 (42.4)	6.74	1.82
1994–1997	34 (16.7)	6.65	1.69	35 (17.1)	7.44	1.87
1998–2000	5 (2.5)	7.68	1.64	5 (2.4)	8.11	1.86
**Maternal age (years)**						
<20	8 (3.9)	6.67	1.57	6 (2.9)	8.65	2.16
20–34	172 (84.7)	6.94	1.81	171 (83.4)	6.74	1.82
≥35	23 (11.3)	6.34	1.64	28 (13.7)	7.05	1.81
**Parity**						
0 [nulliparous]	97 (47.8)	7.00	2.01	106 (51.7)	7.56	2.13
1	71 (35.0)	6.60	1.55	68 (33.2)	6.22	1.55
≥2	35 (17.2)	6.80	1.68	31 (15.1)	6.17	1.42
**Maternal country of origin**						
Sweden	168 (83.3)	7.02	1.85	170 (82.9)	7.06	1.89
Other Nordic countries^a^	7 (3.4)	4.28	2.13	7 (3.4)	6.18	1.69
Rest of Europe^b^	8 (3.9)	7.47	1.60	9 (4.4)	5.48	1.48
Sub-Saharan Africa	2 (1.0)	4.23	0.72	2 (1.0)	2.10	0.45
Middle East and North Africa	13 (6.4)	4.42	0.85	12 (5.9)	2.76	0.47
East Asia	1 (0.5)	6.83	1.71	2 (1.0)	9.36	1.43
South America	2 (1.0)	7.58	2.89	2 (1.0)	7.63	13.5
Unknown	1 (0.5)	2.96	0.46	1 (0.5)	2.60	1.70
**Maternal body mass index (kg/m^2^)** c						
Not available	141 (69.5)	6.85	1.89	142 (69.3)	6.67	1.83
<18.5 (Underweight)	1 (0.5)	10.1	2.64	3 (1.5)	8.75	2.39
18.5–24.9 (Normal)	37 (18.2)	6.83	1.63	42 (20.5)	7.50	1.72
25–29.9 (Overweight)	16 (7.9)	7.27	1.36	14 (6.8)	7.58	2.02
≥30 (Obese)	8 (3.9)	6.06	2.09	4 (2.0)	6.40	2.31
**Smoking during pregnancy** d						
Non-smoker	65 (32.0)	6.54	1.83	85 (41.5)	6.82	1.86
Second-hand smoker	57 (28.1)	7.08	1.71	57 (27.8)	6.91	1.86
Active smoker	81 (39.9)	7.49	1.82	63 (30.7)	6.37	1.72
**Infant sex**						
Male	180 (88.7)	6.97	1.76	163 (79.5)	6.87	1.84
Female	23 (11.3)	6.32	1.99	42 (20.5)	6.51	1.64
**Birth weight (grams)**						
<1500	4 (2.0)	5.73	2.31	0		
<2500	9 (4.4)	4.85	1.44	5 (2.4)	6.37	1.84
2500–4000	166 (81.8)	7.12	1.84	152 (74.1)	6.63	1.82
>4000	24 (11.8)	6.41	1.67	48 (23.4)	7.25	1.94
**Gestational age (weeks)**						
<32	5 (2.5)	4.77	1.44	1 (0.5)	4.71	1.05
<37	6 (3.0)	4.36	1.09	6 (2.9)	4.74	1.97
37–42	176 (86.7)	7.12	1.88	178 (86.8)	6.73	1.82
>42	16 (7.9)	6.54	1.63	20 (9.8)	8.37	1.77
**SD scores (for gestational age)**						
<−2 (small for gestational age)	9 (4.4)	6.69	2.26	14 (6.8)	7.92	1.94
−2 to −1.1	37 (18.2)	6.60	1.53	29 (14.1)	6.56	1.94
−1.1 to 1	138 (68.0)	7.01	1.79	126 (61.5)	6.67	1.77
1.1 to 2	15 (7.4)	5.48	2.00	32 (15.6)	7.79	2.02
>2 (large for gestational age)	4 (2.0)	7.30	2.40	4 (2.0)	4.05	1.20
**Apgar scores**						
0–6	5 (2.5)	4.28	1.80	2 (1.0)	10.2	2.44
≥7	198 (97.5)	6.94	1.79	203 (99.0)	6.74	1.82

Abbreviations: ADHD, attention deficit hyperactivity disorder; PFOS, perfluorooctane sulfonate; PFOA, perfluorooctanoic acid; Parity, number of previous pregnancies.

a Finland, Denmark, and Norway.

b Western Europe and former Eastern Europe.

c Body mass index was classified according to the standard values of the World Health Organization.

d Maternal smoking is based on measured cotinine concentrations in the umbilical cord serum.

**Figure 2:**
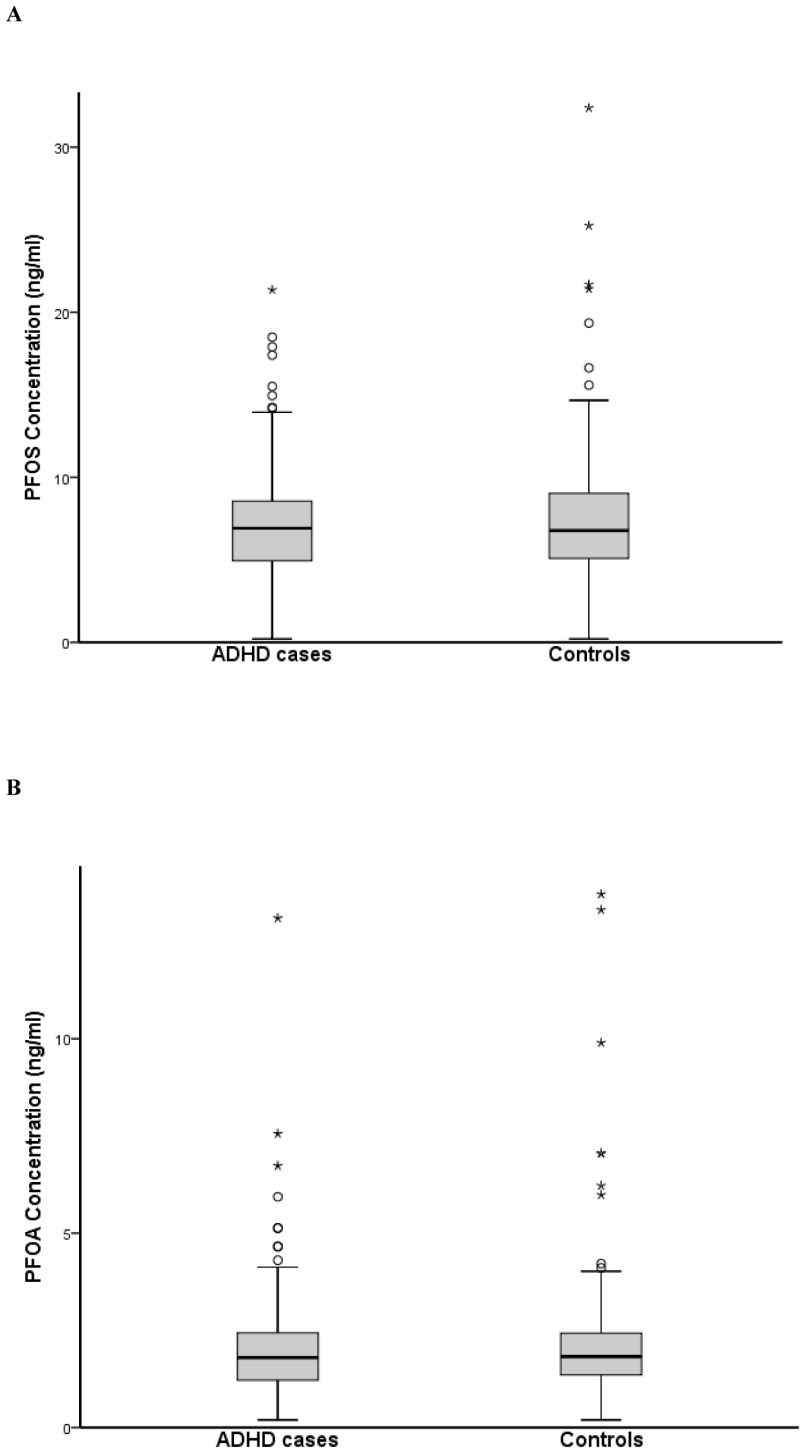
Boxplot of the umbilical cord concentrations of perfluorooctane sulfonate (PFOS) (a) and perfluorooctanoic acid (PFOA) (b) in cases having attention deficit hyperactivity disorder and controls. The extreme values of perfluorooctanoic acid for the ADHD cases, 48 and 36/ml, and for the controls, 66, 49, 31, and 23 ng/ml, are not presented in the boxplot.

Figure 2 shows the distribution of PFOS and PFOA levels in the ADHD cases and the controls. The median concentrations of PFNA above LOD for cases and controls were 0.31 and 0.28 ng/ml, respectively. Wilcoxon's paired test revealed no differences in cord serum PFC concentrations between children with ADHD diagnosis and controls (*p* = 0.72, 0.44, and 0.48 for PFOS, PFOA, and PFNA respectively).

Conditional logistic regression analyses revealed no significant associations between umbilical cord concentrations of PFCs and ADHD (Table 2). The result did not change after adjusting for smoking during pregnancy, parity, and gestational age at birth.

**Table 2:** The crude and adjusted odds ratio with 95% confidence interval of attention deficit hyperactivity disorder and exposure to perfluorinated compounds.

	ADHD Diagnosis	
	Crude	Adjusted^a^
**PFOS** b	0.98 (0.92–1.03)	0.98 (0.92–1.04)
**PFOA** b	0.98 (0.94–1.02)	0.98 (0.94–1.02)
**PFOS** c		
<75^th^ percentile	1	1
≥75^th^ percentile	0.82 (0.51–1.31)	0.81 (0.50–1.32)
**PFOA** c		
<75^th^ percentile	1	1
≥75^th^ percentile	1.03 (0.65–1.6)	1.07 (0.67–1.7)
**PFNA** d		
<LOD	1	1
≥LOD	1.1 (0.72–1.6)	1.1 (0.75–1.7)

Abbreviations: ADHD, attention deficit hyperactivity disorder; PFOS, perfluorooctane sulfonate; PFOA, perfluorooctanoic acid; PFNA, perfluorononanoic acid; LOD, limit of detection.

a Adjusted for maternal active smoking, parity, and gestational age at birth.

b Odds ratio is calculated for 1 ng/ml increase in umbilical cord serum concentration.

c Odds ratio is calculated for PFOS and PFOA concentrations at or above the 75^th^ percentile (75^th^ percentile for PFOS and PFOA were 9,1 ng/ml and 2,4 ng/ml, respectively).

d Odds ratio is calculated for PFNA concentrations at or above the LOD (0.2 ng/ml).

## Discussion

The present study found no statistically significant associations between exposure to PFCs during pregnancy and ADHD diagnosis during childhood, although the measured umbilical cord concentrations of PFOS were among the highest in Europe [42], [48], [49] and even among the highest in the world [9], [50]–[52]. For PFOA, the levels were higher than those measured in Norway and other non-European countries but lower than those in Danmark and Faraoe Islands [9], [42], [48], [49].

Animal data revealed that neonatal mice that were exposed to high doses of PFOS and PFOA showed behavioral defects which ranged from slight effects at the anxiety level [53] to reduced habituation and hyperactivity in adult mice [17]. It has been suggested that PFOS and PFOA act as developmental neurotoxicants that mediate their effects on normal brain development, with consequences for cognitive and behavioral functions, through different mechanisms. Examples of those mechanisms are alteration in the dopaminergic system [17], [18], elevated levels of proteins important for normal neuronal survival, growth and synaptogenesis, such as CaMKII, GAP-43, synaptophysin and tau, in the brain [19], and induction of apoptosis of neuronal cells [54]. Although most of these findings were obtained from experiments on mice or rat derived cell lines that were exposed to extremely high levels of PFOS and PFOA compared to the low levels measured in the present study, other studies found that PFCs were detrimental to neurodevelopment at levels comparable to those observed in humans [17], [19].

Our study is primarily comparable to the study by Fei and Olsen [34] because both studies used measures of prenatal rather than postnatal exposure to PFCs. Fei and Olsen [32] found higher levels of PFOS and PFOA compared to those seen among pregnant women in other countries including the Nordic countries [11], [48]. Consistent with that study, our results provide further indication that fetal exposure to PFCs at the present levels do not play a major role in having ADHD diagnosis at later age. Hoffman et al. [30] found an association between PFC serum concentrations and ADHD in children aged 12 to 15 years. Another study by Stein and Savitz [31] on the relationship between self-reported ADHD and PFC levels in children in the same age range as for those in the study of Hoffman et al. [30] showed an association with PFHxS but not with the other PFCs even though both ADHD prevalence and exposure levels for PFCs were higher in the study by Stein and Savitz. Exposure to PFCs tends to be higher among newborns, toddlers, and children due to high uptake via food consumption, hand-to-mouth transfer of the PFCs from carpets, and through ingestion of dust [55]. If the positive association between PFC exposure and self-reported ADHD found in the study by Hoffman et al. [30] was not due to a chance finding, that might indicate that postnatal exposure to PFCs, rather than prenatal exposure, is associated with ADHD.

The present study has some limitations. Unfortunately, while 419 children with ADHD diagnosis were identified at the Department of Child and Adolescent Psychiatry, there were significant losses to get to the final study sample. The study was restricted to children born in Malmö with available obstetric and demographic information from the SMBR and stored cord blood samples in the biobank. About 50% of the identified children met these two inclusion criteria and were included in the study. The second limitation of the current study is the small number of ADHD cases. Although we would be able to detect an odd ratio of 1.8 and a difference in PFC levels of at least 0.20 standard deviations between cases and controls, it should be emphasized that the statistical power was not high enough to detect some minor associations and we could not accordingly rule out small effects. In addition, we lack information about other exposures that are significant for ADHD, such as exposure to mercury and lead. The PFC levels measured here might also be too low to trigger undesirable effects on the brain development.

During our study period, the clinical diagnostic criteria for ADHD were changed from the definition in DSM-III-R to the definition in DSM-IV, where DSM-IV is regarded as more inclusive. Thus, DSM-IV criteria yield a higher prevalence of ADHD [56]. Most individuals (93%–97.5%) who fulfill a diagnosis of ADHD according to DSM-III-R also fulfill the diagnostic criteria according to DSM-IV [57]-[59]. Individuals with ADHD according to DSM-IV that also fulfilled diagnostic criteria according to DSM-III-R were 85% [57] and 60% [59]. Thus the overlap between ADHD diagnoses according to DSM-III-R and DSM-IV is considerable. The more inclusive diagnosis used in the latter part of the study probably includes some less severe cases which might slightly weaken possible statistical associations between exposure to PFCs and having an ADHD diagnosis.

The study has also several important strengths. First, unlike most of the previous studies on the associations between PFC levels and ADHD, our prospective study design is more reliable in the sense that it is based on clinical diagnosis of ADHD made at the Department of Child and Adolescent Psychiatry. Children were diagnosed at the same psychiatric clinic through the whole study period.

Second, the present study is based on analyzed blood samples from the fetal period, which we believe is the most susceptible exposure window, whereas in other studies, which were of a cross-sectional nature, blood samples were collected from school-age children [30], [31].

Third, we were able to account for important covariates; smoking during pregnancy, parity, and gestational age at birth that have been found to be associated with both PFC levels in pregnant women or infants [11], [38], [42], [46] and ADHD diagnosis or symptoms [39]–[41], [43]–[45]. Epidemiological findings have shown that prevalence of ADHD is higher among males [20]. It has also previously been shown that infant sex has no effect on the concentrations of PFCs [11], [42], [60]. Thus, infant sex was not considered as a potential confounder in the current data set.

In a previous study, we found that fetuses of mothers originating from a country other than Sweden, especially those from Middle East and sub-Saharan Africa, had lower PFC levels in the cord blood than fetuses of native Swedish mothers [11]. Another study found higher odds of having an ADHD diagnosis for native Swedish children compared to children of mothers born outside Sweden [36]. Since the proportion of immigrants is relatively high in Malmö, no matching for the maternal country of birth might result in a false positive relationship between PFCs and ADHD.

Human serum PFC levels showed an increasing pattern from the early 1970s through the late 1990s, followed by leveling out and a decreasing trend right after the phase-out of the production of PFOS and PFOS related compounds in 2002 [8], [61]–[64]. Diagnosis criteria for ADHD have been changed during the study period. We matched for the year of delivery to remove the effect of those differences in PFC levels and diagnosis on the results.

According to our findings, fetal exposure to PFOS, PFOA and PFNA was not associated with ADHD diagnosis in childhood.
